# Roles of serine in neurodegenerative diseases

**DOI:** 10.17179/excli2023-6636

**Published:** 2023-12-08

**Authors:** Jia-Meng Li, Shuang-Qing Zhang

**Affiliations:** 1 Department of Nutrition and Metabolism, National Institute for Nutrition and Health, Chinese Center for Disease Control and Prevention, 27 Nanwei Road, Beijing 100050, China

## ⁯⁯

Neurodegenerative diseases are age-related diseases characterized by cognitive impairment, such as Alzheimer's disease (AD), Parkinson's disease, and schizophrenia (Wu et al., 2022[10]). As a nonessential amino acid, serine plays fundamental roles in neurodegenerative diseases and has two optical isomers of L-serine and D-serine (Zhang and Bai, 2023[11]).

Supplementary L-serine ameliorates cognitive dysfunction in both animals and humans (Handzlik and Metallo, 2023[3]). In the brain, L-serine is predominantly synthesized *de novo *from glucose in astrocytes by 3-phosphoglycerate dehydrogenase due to the low permeability of the blood-brain barrier (BBB) for L-serine, and is indispensable to the biosynthesis of selenoproteins for the maintenance of cognitive functions (Zhang and Bai, 2023[11]). Although the diffusion of D-serine through the BBB still remains slow and weak, its permeability across the BBB is higher than L-serine (Bai et al., 2023[1]). Compared to L-serine, D-serine is a more potent neurotransmitter and a gliotransmitter for neurodegenerative diseases. D-serine is concentrated in the brain, especially the cerebral cortex and hippocampus. As an endogenous amino acid, D-serine is converted from L-serine by pyridoxal 5′-phosphate-dependent enzyme serine racemase (SR) in neurons and astrocytes (Bai et al., 2023[1]), and degraded by D-amino acid oxidase (DAAO) in astrocytes (Ni and Mori, 2022[9]). Overexpression and deficiency of SR are associated with some neurodegenerative diseases, such as AD (Madeira et al., 2015[7]) and schizophrenia (Labrie et al., 2009[5]) since D-serine content depends on SR.

D-serine is a potent co-agonist for N-methyl-D-aspartate glutamate receptor (NMDAR), which plays important pathophysiology roles in synaptic functions, such as synaptic plasticity, learning, and memory. D-serine-mediated NMDAR activation is crucial for the regulation of neurodegenerative diseases, however, the overactivation of NMDAR induces excitotoxicity, thus leading to cognitive impairment (Mota et al., 2014[8]). D-serine is considered as a biomarker of neurodegenerative diseases because low D-serine levels were observed in AD animals and patients (Le Douce et al., 2020[6]; Madeira et al., 2015[7]). However, D-serine was not significantly different in cerebrospinal fluid between schizophrenic patients and healthy controls (Fuchs et al., 2008[2]). Oral D-serine supplementation for 2 weeks restored the spatial memory deficits in transgenic AD mice (Le Douce et al., 2020[6]), and oral 4-week D-serine at doses of 60 and 120 mg/kg/day effectively improved persistent symptoms and neurocognitive dysfunction in schizophrenic patients (Kantrowitz et al., 2010[4]). 

Taken together, serine shows promising potential in the prevention and treatment of neurodegenerative diseases, however its exact mechanism remains unclear. Further studies are needed to confirm the mechanism.

## Declaration

### Conflicts of interest

The authors declare that there are no conflicts of interest.

### Acknowledgments

None.

### Funding

The authors declare that no funds, grants, or other support were received during the preparation of this letter.
